# A novel *SLC26A4* splicing mutation identified in two deaf Chinese twin sisters with enlarged vestibular aqueducts

**DOI:** 10.1002/mgg3.1447

**Published:** 2020-08-07

**Authors:** Kai Zhou, Lancheng Huang, Menglong Feng, Xinlei Li, Yi Zhao, Fei Liu, Jiazhang Wei, Danxue Qin, Qiutian Lu, Min Shi, Shenhong Qu, Fengzhu Tang

**Affiliations:** ^1^ Department of Otolaryngology & Head and Neck The People's Hospital of Guangxi Zhuang Autonomous Region Nanning China; ^2^ Guangxi Medical University Nanning Guangxi China; ^3^ Guangxi University of Chinese Medicine Nanning Guangxi China; ^4^ Medical Genetics Center Southwest Hospital Army Medical University (Third Military Medical University) Chongqing China; ^5^ Research Center of Medical Sciences The People’s Hospital of Guangxi Zhuang Autonomous Region Nanning China

**Keywords:** c.1614+5G>A, enlarged vestibular aqueduct, minigene, *SLC26A4* gene

## Abstract

**Background:**

Variants in the *SLC26A4* gene are correlated with nonsyndromic hearing loss with an enlarged vestibular aqueduct (EVA). This study aimed to identify the genetic causes in a Chinese family with EVA, and the pathogenicity of the detected variants.

**Methods:**

We collected blood samples and clinical data from a pair of deaf twin sisters with EVA and their family members. As controls, a group of 500 normal‐hearing people were enrolled in our study. Twenty‐one exons and flanking splice sites of the *SLC26A4* gene were screened for pathogenic mutations by polymerase chain reaction and bidirectional Sanger sequencing. Minigene assays were used to verify whether the novel *SLC26A4* intronic mutation influenced the normal splicing of mRNA.

**Results:**

Hearing loss in the twins with EVA was diagnosed using auditory tests and imaging examinations. Two pathogenic mutations, c.919‐2A>G and c.1614+5G>A were detected in *SLC26A4*, the latter of which has not been reported in the literature. The minigene expression *in vitro* confirmed that c.1614+5G>A could cause aberrant splicing, resulting in skipping over exon 14.

**Conclusions:**

On the *SLC26A4* gene, c.1614+5G>A is a pathogenic mutation. This finding enriches the mutational spectrum of the *SLC26A4* gene and provides a basis for the genetic diagnosis of EVA.

## Graphical abstract

We found a novel SLC26A4 splicing mutation in two deaf Chinese twin sisters with enlarged vestibular aqueducts. Then, mini‐gene assays were used to verify whether the novel SLC26A4 intronic mutation influenced the normal splicing of mRNA. Finally, we confirmed that the novel SLC26A4 splicing mutation, c.1614+5G>A, is a pathogenic mutation.

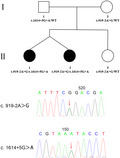

## 1 INRTODUCTION

Hearing loss is one of the most common sensory defects, with a significant impact not only on quality of life, but also on the physical and mental well‐being of the affected individuals (Brown, Emmett, Robler, & Tucci, 2018). According to the 2015 Global Burden of Disease Study, half a billion people suffer from disabling hearing loss worldwide. The social and economic burden is, therefore, significant (GBD, 2015 Disease and Injury Incidence and Prevalence Collaborators. Global, regional, and national incidence, prevalence, and years lived with disability for 310 diseases and injuries, 1990–2015: a systematic analysis for the Global Burden of Disease Study 2015, 2016). Genetic causes likely account for most cases worldwide. Mutations can impact nearly all components of the auditory pathway; however, inner ear homeostasis and mechano‐electrical transduction are particularly vulnerable (Cryns & Van Camp, 2004; Korver et al., 2017). Many people with hearing loss have malformations of the inner ear, and an enlarged vestibular aqueduct (EVA) is one of the most frequent inner ear malformations related to sensorineural hearing loss (SNHL) in children (Griffith & Wangemann, 2011). Most patients with EVA present with nonsyndromic hearing loss, a small number of which are complicated by a goiter, called Pendred syndrome (PDS) (Wemeau & Kopp, 2017). EVA syndrome (EVAS) is a common type of autosomal recessive hearing loss (Wemeau & Kopp, 2017). A patient is diagnosed with EVA if the midpoint between the common crus and the external aperture measures more than 1.5 mm, and it is detectable in the inner ears of patients with PDS by computed tomography (CT) or magnetic resonance imaging (MRI) (Phelps et al., 1998). Studies have shown that biallelic mutations in the *SLC26A4* (OMIM 605646) gene are the main cause of EVA (Usami et al., 1999). The *SLC26A4* gene is located on human chromosome 7q31 and encodes a 780‐amino‐acid (86 kD) transmembrane anion transport protein, pendrin (Everett et al., 1997), which is expressed in the thyroid, kidney, and cochlea (Everett, Morsli, Wu, & Green, 1999). In the inner ear, pendrin is thought to modulate Cl^−^/HCO_3_^−^ exchange and is therefore responsible for the conditioning of endolymphatic fluid, presumably due to HCO3^−^ secretion (Wangemann et al., 2007). The mutation c.919‐2A>G is the most frequent hot‐spot mutation of the *SLC26A4* gene in the Chinese deaf population, and it is the second most common mutation in other Asian countries (Cheng et al., 2019). Guangxi is an area inhabited by ethnic minorities, mainly the Zhuang people. Our previous research showed that the rate of hotspot mutations in common genes correlated with deafness (*GJB2*,* GJB3*,* 12S rRNA*, and *SLC26A4*) in the Guangxi region was approximately 9%, which was much lower than the rate in a large cohort of the Chinese population (Liu et al., 2015).

Here, we report the case of a pair of twin sisters with profound sensorineural hearing loss at our department in Guangxi Zhuang Autonomous Region, China. Through CT imaging of the temporal bone or an MRI of the inner ear, bilateral EVAs and endolymphatic sacs were observed in the two sisters’ inner ears. To investigate the genetic causes, screening of common deafness genes (4 genes and 15 pathogenic variants) was performed on this family using a microarray method with a hereditary deafness gene mutation detection kit (CapitalBio Technology Co., Ltd. Beijing, China). The results showed that the twin sisters, their mother, and the third sister all carried the *SLC26A4* c.919‐2A>G mutation, whereas no mutations were detected by the microarray method in the father. Subsequently, all exons and flanking splice sites of the *SLC26A4* gene in these family members were screened for mutations using polymerase chain reaction (PCR) amplification and bidirectional sequencing. Interestingly, we were then able to identify that the deaf twin sisters carried the novel splicing site c.1614+5G>A mutation inherited from their normal‐hearing father. The c.1614+5G>A mutation is not mentioned in ClinVar, DVD, PubMed, or HGMD and has never been described in clinical reports. To determine whether this site is a pathogenic site for the affected twin sisters, we performed a *SLC26A4* gene c.1614+5G>A mutation pathogenicity study.

## 2 MATERIALS AND METHODS

### 2.1 Subjects

This study was approved by the Ethics Committee of the People's Hospital of Guangxi Zhuang Autonomous Region. The study subjects were deaf twin sisters with cochlear implantations and their family members at the Department of Otolaryngology Head and Neck Surgery of the People's Hospital of Guangxi Zhuang Autonomous Region. The parents and the third sister in the family had normal hearing, while the twin sisters had profound sensorineural hearing loss. We also collected blood and clinical audiology data from 500 control subjects with normal hearing. Written informed consents were obtained from the participants or their parents.

### 2.2 Audiological and imaging evaluation

All subjects underwent a physical examination, including an otoscopic examination, with special attention to hearing. The comprehensive audiological evaluation included auditory brainstem response (ABR), distortion product otoacoustic emission (DPOAE), auditory steady‐state response (ASSR), and pure tone audiometry (PTA).

A CT of the temporal bone or an MRI of the inner ear was performed.

### 2.3 DNA extraction

Genomic DNA was extracted from a 3‐5 ml whole blood sample taken from five members of the deaf twin sisters’ family and 500 people with normal hearing. The steps were performed according to the manufacturer's instructions (Tiangen Biotech, Beijing, China).

### 2.4 Mutation screening

Common deafness gene screenings (4 genes and 15 pathogenic variants) were performed on these family members using the microarray method with a hereditary deafness gene mutation detection kit (CapitalBio Technology Co., Ltd. Beijing, China). The results indicated that the twin sisters, their mother, and the third sister carried the *SLC26A4* (NM_000441.1) c.919‐2A>G mutation, while there was no mutation detected in the father at any mutation site. Subsequently, all exons and flanking splice sites of the *SLC26A4* gene in these family members were screened for mutations by PCR amplification and bidirectional sequencing. We were then able to discover the c.1614+5G>A mutation site derived from the father that the deaf twin sisters also carried. All exons and flanking splice sites of the *SLC26A4* gene in the 500 normal‐hearing participants were then screened for mutations by PCR amplification and bidirectional sequencing to analyze any *SLC26A4* c.1614+5G>A mutation carriers.

### 2.5 *In silico* analysis

The bioinformatics splicing tool Human Splicing Finder (HSF) version 3.0 (http://www.umd.be/HSF3/), and fruit fly (http://www.fruitfly.org/) software were used to predict the possible influence of this mutation in the intron.

### 2.6 Construction of recombinant plasmids

Three rounds of PCR were performed using nested primers: the first PCR was performed using genomic DNA (a total of two sets of DNA) as a template, with SLC26A4‐36511‐F and SLC26A4‐38609‐R as primers for 30 cycles; the second PCR was performed using products from the first round of PCR as a template, with SLC26A4‐36999‐F and SLC26A4‐38154‐R as primers for 30 cycles; the third PCR was performed using PCR products from the second round as a template, with pcMINI‐SLC26A4‐BamHI‐F and pcMINI‐SLC26A4‐EcoRI‐R as primers for 30 cycles (genomic DNA, gene names and primers are shown in Tables 1 and 2). Electrophoresis and gel recovery of the last round of PCR products were then performed. Both pcMINI‐SLC26A4‐wt (wild‐type) and pcMINI‐SLC26A4‐mut (c.1614+5G>A) contained the entire sequence of exon 14 and part of the upstream and downstream introns, and the amplified length was 764 bps.

**Table 1:** Genomic DNA and gene names corresponding to each group

Genomic DNA	Name of gene
(1) Control group	pcMINI‐SLC26A4‐wt (Wild‐type)
(2) Experimental group (proband)	pcMINI‐SLC26A4‐mut (c.1614+5G>A)

**Table 2:** Primers used in this study

Primers	Sequence 5′→3′	Melts degree	Product length
SLC26A4‐36511‐F	agatgctgtctctcatgctg	57°C	2099 bp
SLC26A4‐38609‐R	ggctgaatgacttgggcaaa		
SLC26A4‐36999‐F	ctctgctgccctttacctcc	56°C	1156 bp
SLC26A4‐38154‐R	aggtcttggtcatagagaac		
pcMINI‐SLC26A4‐BamHⅠ‐F	gctcggatcccagggttatggcagggctta	57°C	764 bp
pcMINI‐SLC26A4‐EcoRⅠ‐R	tgcagaattccctgcctgtaatcccagcca		

The PCR products pcMINI‐SLC26A4‐wt (wild‐type) and pcMINI‐SLC26A4‐mut (c.1614+5G>A) were purified and inserted into the eukaryotic expression vector pcMINI using BamHI/EcoRI to construct two sets of plasmids: pcMINI‐SLC26A4‐wt and pcMINI‐SLC26A4‐mut. The recombinant plasmids were digested using BamHI and EcoRI and verified by gene sequencing.

### 2.7 Transfection of eukaryotic cells

The recombinant vectors (pcMINI‐SLC26A4‐wt and pcMINI‐SLC26A4‐mut) were transiently transfected into human embryonic kidney cells (HEK‐293T) and cervical cancer cells (HeLa) according to the manufacturer's instructions. The transfected cells were cultured for 36 h and then collected for analysis.

### 2.8 Reverse transcription‐polymerase chain reaction

All the RNA was extracted from 293T and HeLa cells using the Trizol method (RNA extraction kit, Omega). RNA concentrations and purity were assessed using UV spectrophotometry, and the cDNA was synthesized using a reverse transcription kit (Thermo). PCR products were identified using 2% agarose gel electrophoresis and verified through sequencing.

## 3 RESULTS

### 3.1 Analysis of clinical manifestations and imaging data

**Figure 1:**
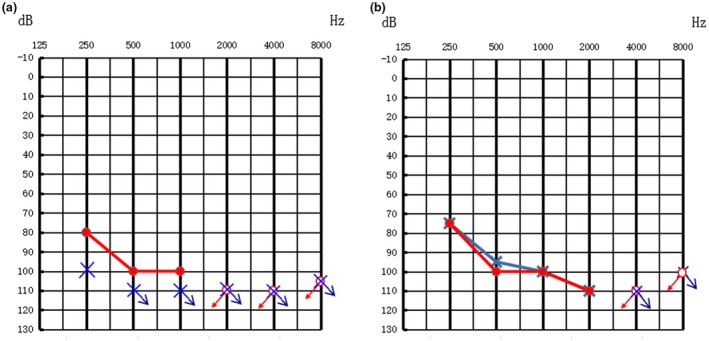
Audiograms of II:1 and II:2. (a) The squares in red represent the right ear; diamonds in blue represent the left ear (b) The squares in red represent the right ear; diamonds in blue represent the left ear

**Figure 2:**
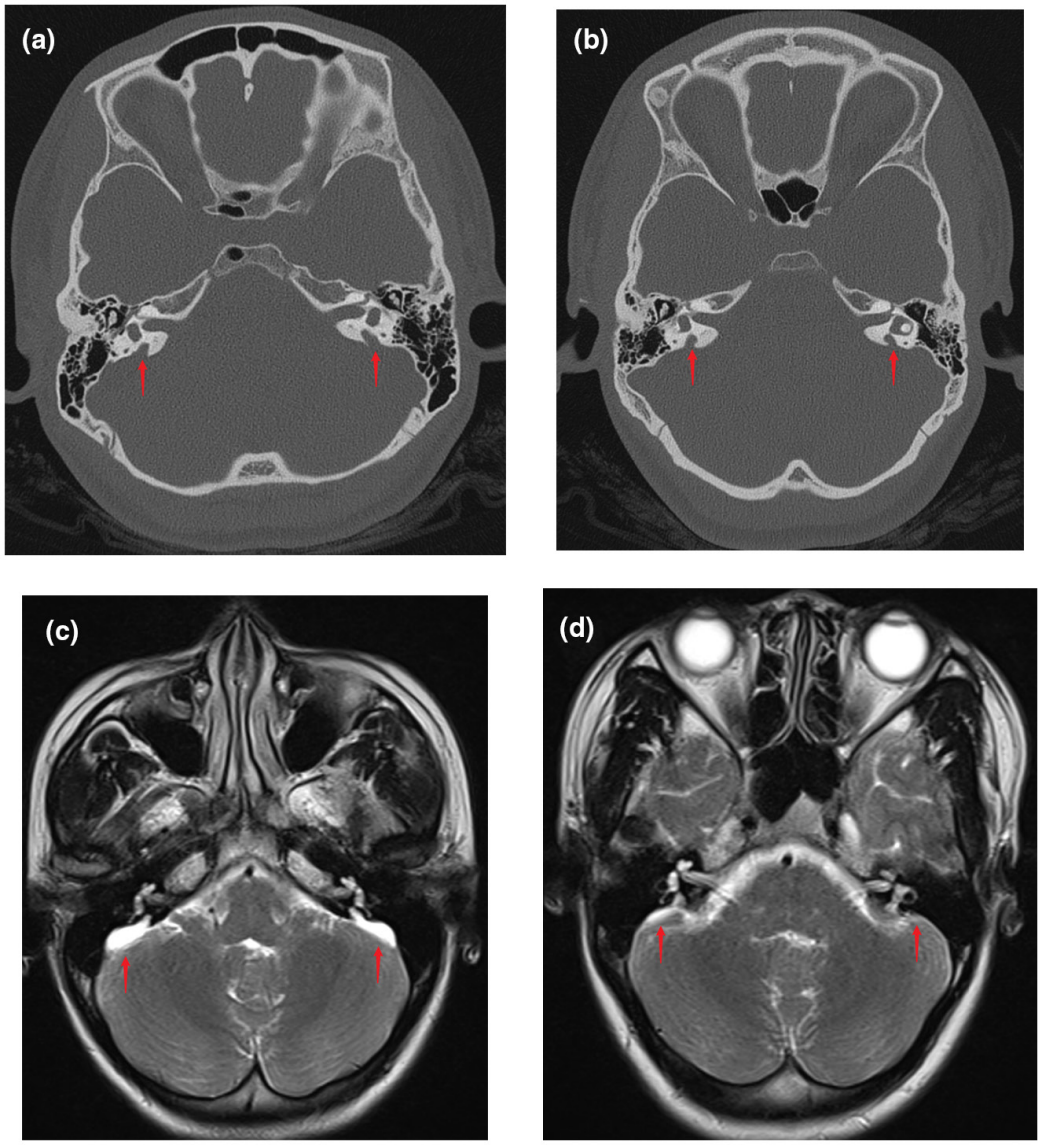
Imaging examinations of II:1 and II:2. (a) and (b) are CTs of the temporal bone of II:1 and II:2, respectively (red arrows denote the location of the enlarged vestibular aqueduct). (c) and (d) show MRIs of the inner ear of II:1 and II:2, respectively (red arrows denote the location of the enlarged endolymphatic sac)

All the subjects were negative for systemic and thyroid disease, and the comprehensive physical examinations and otoscopies were also normal. The deaf twin sisters both had thresholds of ABR air conduction that were 90 dB nHL on the right and 95 dB nHL on the left side. They had bilateral type "A" tympanograms, and bilateral acoustic stapedial reflexes were not elicited. DPOAE did not elicit any response from the patients in either ear. PTA demonstrated that the affected twin sisters had profound sensorineural hearing impairment (Figure 1a,b), whereas the hearing of the parents and the third sister were normal. CTs of the temporal bones and MRIs of the inner ears of the deaf twin sisters revealed bilateral EVAs and endolymphatic sac enlargement (Figure 2a–d). The deaf twin sisters were diagnosed with bilateral EVAS in our hospital according to the CT. The CTs of the temporal bones were normal for both parents and the third sister.

### 3.2 Genetic analysis

**Figure 3:**
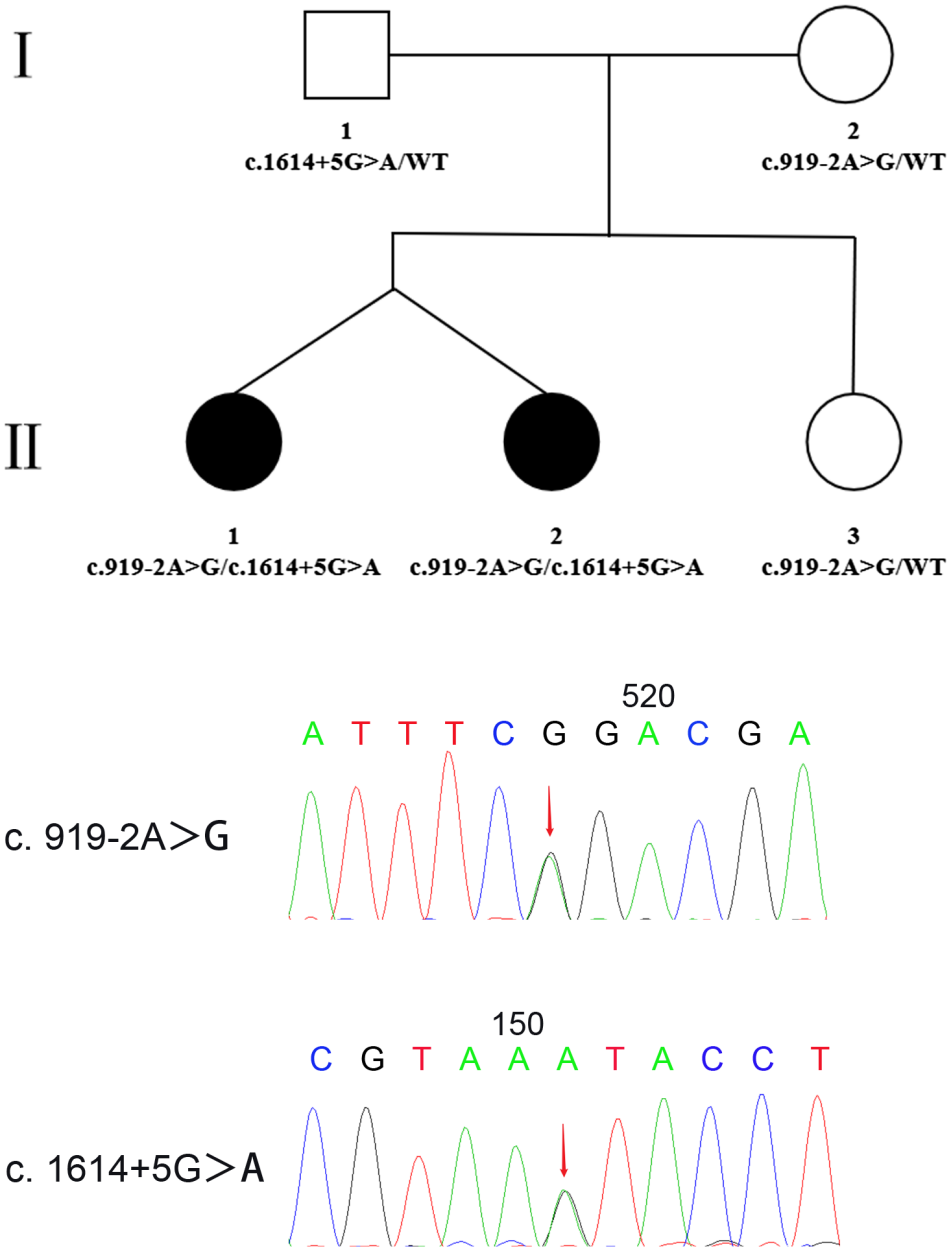
Family's pedigree and genotype are shown. Filled symbols represent affected individuals. The c.919‐2A>G and c.1614+5G>A mutations, both present in family members II:1 and II:2, are indicated by red arrows

Sanger sequencing of the *SLC26A4* gene demonstrated that the twin sisters in this family had compound heterozygous mutations of c.919‐2A>G (IVS7‐2A>G) (rs111033313) in intron 7 and c.1614+5G>A in intron 14. In addition, the father was a heterozygous carrier of the c.1614+5G>A mutation, and the mother and the third sister were heterozygous carriers of the c.919‐2A>G mutation (Figure 3). The *SLC26A4* c.1614+5G>A mutation site was detected by PCR and Sanger sequencing in the 500 normal‐hearing controls. The results showed that the c.1614+5G>A variant was not detected in our controls and was as low as 0.003186% when compared to GnomAD (http://gnomad.broadinstitute.org).

### 3.3 *In silico* splicing analysis

According to the fruit fly analysis, the fifth base guanine of intron 14 was mutated to adenine. The confidence scores of the donor splicing site were 0.99 to 0.83 before and after the mutation, respectively, which was a significant change. HSF predicted that the mutation alters the wild‐type donor splicing site and is likely to affect the splicing of mRNA.

### 3.4 *SLC26A4* mRNA expression in cells transfected with recombinant plasmids

**Figure 4:**
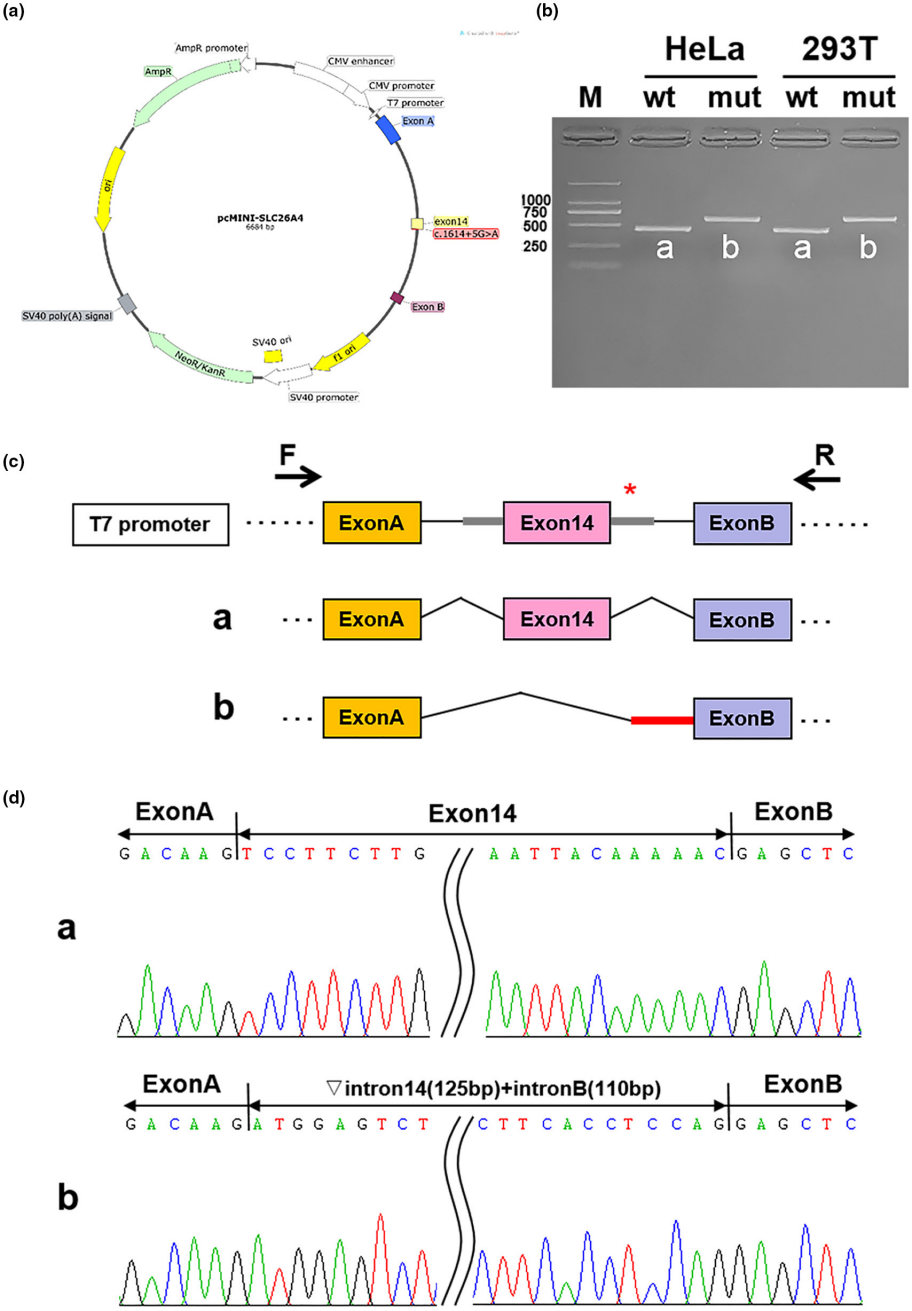
Splicing alteration identified using a minigene assay. (a) Construction of the pcMINI‐SLC26A4‐wt/mut vector harboring exon 14 and flanking intronic sequences from wild‐type or variant types (c.1614+5G>A) of the *SCL26A4* gene. (b) Reverse‐transcription polymerase chain reaction (RT‐PCR) products were separated by electrophoresis of the pcMINI‐SLC26A4‐wt/mut vector in HeLa and c293T cells. The different splicing products for wild‐type (wt lane, 459 bps) and variant type (mut lane, 694 bps) are shown on 2% agarose gel electrophoresis and represented graphically. (c) Schematic diagram of minigene construction and schematic diagram of Sanger sequencing of RT‐PCR products. (d) Sequencing results for the bands

A minigene splicing assay was performed to validate whether this variant affects splicing products. HeLa and 293T cells were transfected as described above. A total of 4 samples were collected after 36 h of transfection. A schematic diagram of the minigene construct is shown in Figure 4a.

The results of the reverse transcription‐PCR showed that the band of the mutant‐type (mut) was larger than that of the wild‐type (wt), and the migration was slower (Figure 4b). DNA sequencing results indicated that the wild‐type minigene (pcMINI‐SLC26A4‐wt) formed normal mRNA composed of exon 14 (Figure 4c,d). The intron c.1614+5G>A mutant minigene caused aberrant splicing, resulting in the skipping of exon 14 and the retention of the 235 bp base in intron 14 and intron B (Figure 4c,d). This result is consistent with the HSF analysis result.

## 4 DISSUSSION

Two mutations on the *SLC26A4* gene of the deaf twin sisters in this study were detected: one in intron 7 (c.919‐2A>G) from the mother; and one in intron 14 (c.1614+5G>A) from the father. Although intronic variants may have more harmful effects than exonic variants, they are rarely studied (Kallel‐Bouattour et al., 2017). To further clarify the pathogenicity of intronic mutation c.1614+5G>A, we performed reverse transcription‐PCR splicing validation by constructing the minigene vector. The results showed that the intron c.1614+5G>A mutation caused aberrant splicing, resulting in the skipping of exon 14 and the retention of the 235 bp base in intron 14 and intron B (Figure 4d). This variation, therefore, would lead to either exon skipping or intron retention, which would consequently result in an altered protein structure (Tang et al., 2016).

It has been identified that the c.919‐2A>G mutation is situated at the 3’ splice site of exon 8. Transcripts of the *SLC26A4* gene with this mutation skip exon 8 entirely, resulting in a new connection between exon 7 and exon 9 (Wang et al., 2007). The variant c.1614+5G>A is also an intronic region mutation that has not been reported before. Splicing variants in this domain have been associated with EVA and Pendred syndrome (Table 3). As suggested by the ACMG/AMP guidelines and the expert specification of variant interpretation guidelines for genetic hearing loss (Alford et al., 2014; Oza et al., 2018; Richards et al., 2015), a pathogenicity analysis was performed: (a) validation of the *in vitro* experiment was shown through constructing the minigene vector, which demonstrated that the intron c.1614+5G>A mutation caused aberrant splicing, which subsequently would lead to gene function impairment (strong pathogenic evidence PS3); (b) the novel variant *SLC26A4* c.1614+5G>A mutation was not identified in our controls and had an incidence as low as 0.003186% when compared to GnomAD (moderate pathogenic evidence PM2) (Lek et al., 2016); (c) EVAS is a recessive disorder and we identified a pathogenic variant, c.919‐2A>G; therefore, the second allele in the deaf twin sisters is considered evidence of pathogenicity (moderate evidence PM3); (d) the deaf twin sisters’ phenotype, hearing loss, and EVA are highly specific to the *SLC26A4* gene (supporting pathogenic evidence, PP4); and (e) the affected twins were found to carry the *SLC26A4* c.919‐2A>G and c.1614+5G>A compound heterozygous mutations. The normal‐hearing individuals in the family only had one mutant allele in the *SLC26A4* gene (the father carried the *SLC26A4* c.1614+5G>A mutation, and the mother and the third sister carried the *SLC26A4* c.919‐2A>G mutation), confirming that the phenotype of hearing loss and EVA was co‐segregated with genotype in this family (supportive pathogenic evidence, PP1). Taken together, the evidence for the c.1614+5G>A mutation is “PS3+PM2+PM3+PP4+PP1” and is judged to be a pathogenic mutation (very strong pathogenic evidence).

**Table 3:** Genotypic and phenotypic information on mutation sites reported near this site

Genotypic	Phenotypic
c.1614+1G>A	Pendred syndrome
c.1614+1G>C	Pendred syndrome
c.1614+1G>T	Enlarged vestibular aqueduct
c.1614+7A>G	Pendred syndrome
c.1615‐2A>G	Pendred syndrome
c.1615‐1G>A	Enlarged vestibular aqueduct
c.1615‐1G>C	Enlarged vestibular aqueduct

*SLC26A4* mutations are the second‐most common cause of deafness in the Chinese mainland deaf population. The incidence of *SLC26A4* gene mutations accounts for 20.35%, ranking only second to *GJB2* (25.65%) (Yu et al., 2020). The incidence rate of EVA in the Chinese deaf population was found to be at least 11% (Yuan et al., 2012). The *SLC26A4* gene is located on human chromosome 7q31, contains 21 exons, and produces a transcript of approximately 5 kb. The translated protein, pendrin, is closely related to a number of sulfate transporters (Royaux et al., 2001). Due to the length of the *SLC26A4* gene sequence and abundant introns, currently 4208 variants in this gene have been reported, of which 420 have been identified as pathogenic mutations. There are 86 splice site mutations, accounting for approximately 20.5% of all pathogenic sites (http://deafnessvariationdatabase.org/, accessed on May 10, 2020). Based on these results, we propose that mutations of 10 bps at exon‐intron boundaries are most likely to be pathogenic. At present, there have only been a few functional experiments on deafness gene mutation sites. Therefore, *in silico* predictions can provide an effective reference.

In this family, a pair of twin sisters had profound sensorineural hearing loss. Through CT imaging of the temporal bone or an MRI of the inner ear, the twin sisters were shown to have bilateral EVA, and the bilateral thyroid gland was not palpable. Auditory evaluations demonstrated bilateral profound sensorineural hearing loss on PTA, ABR, DPOAE, and ASSR. Therefore, we diagnosed the affected twin sisters with EVAS. *SLC26A4* gene mutations mainly cause cochlear dysfunction and inner ear dysplasia and do not alter the polarity sensitivity of the auditory nerve fibers to electrical stimulation (Luo et al., 2020; Wangemann, 2013). Some studies have indicated that cochlear implants should be effective in patients with *SLC26A4* gene mutations (Lai et al., 2012; Wu et al., 2015; Yan, Li, Yang, Huang, & Wu, 2013). Therefore, these twin sisters received single‐sided cochlear implantation at the age of 20 years at our hospital. They had cerebral spinal fluid gushers on cochleostomy, but both patients subsequently had full electrode insertions with no further problems. After one year of rehabilitation, we evaluated the twins’ hearing and speech function. They had nearly normal hearing and could communicate with simple words.

## 5 CONCLUSIONS

In this study, *SLC26A4* c.1614+5G>A was found to be a pathogenic mutation. The novel finding enriches the mutant spectrum of the *SLC26A4* gene and provides a basis for genetic counseling and diagnosis of hearing loss with EVA. Using the banding microarray method with a hereditary deafness gene mutation detection kit and sequencing analysis is an effective and high‐throughput approach for the diagnosis of genetic hearing impairment.

## CONFLICT OF INTEREST

The authors declare that they have no competing interests.

## AUTHOR CONTRIBUTIONS

KZ, DXQ, and MS carried out the mini‐gene assays and Sanger sequence. YZ, KZ, XLL, and FL carried out the molecular genetic studies and the bioinformatic analysis of the sequencing data. KZ drafted the paper. JZW, FZT, and SHQ given final approval of the version to be published FZT, SHQ, and QTL conceived the study, participated in its design and coordination. All authors have read and approved the final paper.
